# Prenatal GABAB Receptor Agonist Administration Corrects the Inheritance of Autism-Like Core Behaviors in Offspring of Mice Prenatally Exposed to Valproic Acid

**DOI:** 10.3389/fpsyt.2022.835993

**Published:** 2022-04-15

**Authors:** Shucai Jiang, Maotao He, Lifei Xiao, Yu Sun, Jiangwei Ding, Wenchao Li, Baorui Guo, Lei Wang, Yangyang Wang, Caibin Gao, Tao Sun, Feng Wang

**Affiliations:** ^1^Ningxia Key Laboratory of Craniocerebral Disease, Incubation Base of National Key Laboratory, Ningxia Medical University, Yinchuan, China; ^2^School of Basic Medical Sciences, Weifang Medical University, Weifang, China; ^3^The First Affiliated Hospital, Zhejiang University School of Medicine, Hangzhou, China

**Keywords:** autism spectrum disorder (ASD), valproic acid (VPA), baclofen, GABAB, inheritance, mice

## Abstract

This study was performed to evaluate the effects of prenatal baclofen (a GABAB receptor agonist) treatment on the inheritance of autism-like behaviors in valproic acid (VPA)-exposed mice. VPA model mice (first generation, F1) that were prenatally exposed to VPA exhibited robust core autism-like behaviors, and we found that oral administration of baclofen to F1 mice corrected their autism-like behavioral phenotypes at an early age. Based on a previous epigenetics study, we mated the F1 male offspring with litter females to produce the second generation (F2). The F2 male mice showed obvious inheritance of autism-like phenotypes from F1 mice, implying the heritability of autism symptoms in patients with prenatal VPA exposure. Furthermore, we found prenatal baclofen administration was associated with beneficial effects on the autism-like phenotype in F2 male mice. This may have involved corrections in the density of total/mature dendritic spines in the hippocampus (HC) and medial prefrontal cortex (mPFC), normalizing synaptic plasticity. In this research, GABAB receptor agonist administration corrected the core autism-like behaviors of F1 mice and protected against the inheritance of neurodevelopmental disorders in the offspring of F1 mice, suggesting the potential of early intervention with GABAB receptor agonists in the treatment of neurodevelopmental disorders.

## Introduction

Autism is a lifelong neurodevelopmental disease and one of the most serious developmental psychiatric disorders known today; in 2013, the diagnoses of “autism” and several other disease categories were incorporated into the single diagnostic category “autism spectrum disorder” (ASD) (Diagnostic and Statistical Manual of Mental Disorders, DSM-5) (1, 2). The new diagnostic criteria include social/communication deficits (criterion A) and restricted, repetitive patterns of behaviors, interests, or activities (criterion B). These symptoms are present beginning in early childhood (criterion C) and limit or impair daily life (criterion D) (2, 3).

In recent years, the incidence of ASD has increased persistently (4, 5). Based on its complex pathophysiological mechanism and lack of effective drug treatments, ASD has attracted attention worldwide (6–9). While autism has a strong genetic component, environmental factors, including intrauterine exposure to drugs, toxins, pesticides, and infection, are known to confer susceptibility to autism (6, 10, 11). Accumulating clinical evidence suggests that prenatal exposure to the anti-epileptic drug valproic acid (VPA) is associated with an increased risk of ASD, neurodevelopmental delay, and cognitive deficits in children (10–12). Consistent with clinical evidence, rodents prenatally exposed to VPA exhibit behavioral deficits resembling autism-like symptoms (12).

Animal studies have shown that *in utero* exposure to VPA in rodents represents a robust model of autism that exhibits face, construct and predictive validity (6, 11). This model has been widely used in preclinical research to reveal the etiology of environmental factors contributing to ASD and identify new drug treatment targets (12). Studies have found that VPA rodent models exhibit robust behavioral changes and molecular pathology involving dysfunctional GABAergic signaling, extensive alterations in neuronal morphology and local neocortical microcircuit disruption (13–22). Dysregulation of the GABAergic system and excitatory-inhibitory (E-I) imbalance have commonly been observed in rodent models of autism (16, 23), and correction of these changes with pharmacological interventions normalizes core autism-like phenotypes in these animals (24, 25). According to recent studies, the autism-like symptoms in a genetically defective mouse model of ASD were corrected by the GABAB2 receptor agonists baclofen and arbaclofen (STX209, an exploratory drug comprising the single, active R-enantiomer of baclofen) (26–30). Although the results of the majority of clinical trials also supported the therapeutic effect of R-baclofen (31–35), the phase 3 clinical trial of arbaclofen for the treatment of patients with fragile X syndrome presenting the ASD phenotype was prematurely terminated due to lack of efficacy (but the highest dose exerted a beneficial effect on treated children) (36). Hence, we infer that GABAB receptor agonists are effective treatments for some but not all subgroups of patients with ASD. The possible therapeutic effect of GABAB agonists on children with ASD caused by environmental factors (such as VPA) aroused our attention. In addition, there are no relevant reports have assessed oral baclofen administration in mice/children prenatally exposed to VPA. Part of the present study showed that long-term oral treatment with baclofen attenuated autism-like behaviors in young F1 mice, suggesting that the GABAB receptor agonist treatment could correct the core autism-like symptoms in mice prenatally exposed to VPA.

Additionally, ASD is also accompanied by a high risk of transmission to the next generation (37–40), and epigenetics may play an important role in this process (41–43). Studies have verified that epigenetics might be implicated in the mechanisms underlying neurodevelopmental disorders caused by VPA (10–12, 44). Recent research showed that the offspring of male mice prenatally exposed to VPA (F1) mated with control female mice transmit the autism-like phenotype to the F2 and F3 generations (45, 46), suggesting that prenatal exposure to VPA causes autism-like symptoms with strong heritability. Meanwhile, a report showed that pregabalin (a GABA analog) administered during the pregnancy period could correct autism-like behavioral defects in rats prenatally exposed to VPA (47). Based on the above results, prenatal intervention with baclofen in the offspring (F2) of VPA-exposed mice (F1) with autism-like behaviors aroused our interest.

The results indicated that (1) the offspring produced by mating within the same litter of F1 mice exhibited obvious autism-like behaviors, suggesting that the autism-like symptoms of F1 mice were inherited by the next generation; and (2) prenatal administration of baclofen corrected autism-like symptoms in F2 mice by correcting the defects in the density of total/mature dendritic spines in the hippocampus (HC) and medial prefrontal cortex (mPFC). The morphology and density of dendritic spines play crucial functional roles in synaptic plasticity and, consequently, in learning and memory processes (48). Therefore, the activation of the GABAergic pathway may exert beneficial and profound effects on early brain development in F2 mice.

## Materials and Methods

### Animals

Breeding pairs of C57BL/6J mice were purchased from Ningxia Medical University Laboratory Animal Center (Ningxia, China) and housed in a conventional mouse vivarium at the Feeding Unit of Ningxia Medical University Craniocerebral Laboratory (Ningxia, China). Each male mouse was housed in a single cage, and female mice were housed in groups of 2 (random allocation). Standard rodent chow and tap water were available ad libitum. All mice were maintained under standard laboratory conditions at 22 ± 22°C with 50 ± 10% relative humidity and a 12-h light/dark cycle. For the present experiments, a total of 67 male offspring from 22 dams have been used.

#### Breeding Process

Breeding pairs mice aged ~10 weeks, with female mice weighing 20–25 g and male mice weighing 22–25 g. Precontact between male and female mice was established for 3 days to regulate the fertility cycle, and when the females were in a proestrus state, the animals were allowed to mate overnight, namely, from 5 p.m. to 8 a.m. the next day. Detection of a vaginal plug in female mice was designated 0.5 days of pregnancy. Because of the precontact procedure, female pregnancy and pup birth occurred within a 3-day period. All mice were handled according to protocols approved by the Institutional Animal Care and Use Committee of Ningxia Medical University (IACUC Animal Use Certificate No. 2019-152). All efforts were made to minimize the number of animals used and their suffering.

#### Prenatal VPA Exposure

**Figure 1:**
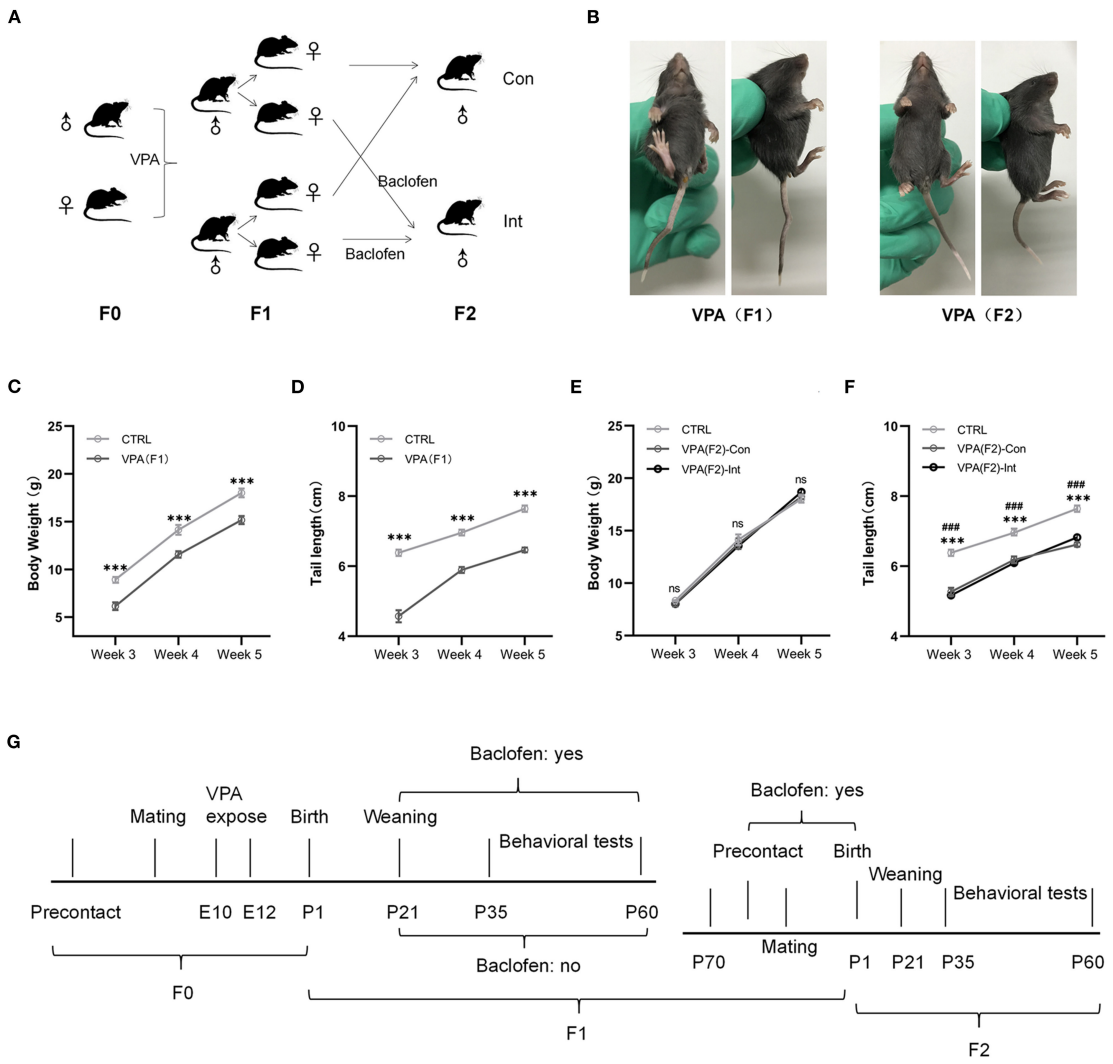
F1 mice exhibited delayed growth and tail malformations, and F2 mice exhibited delayed tail growth without malformation. **(A)** The schematic diagram of F1 and F2 mice breeding. **(B)** The pictures of F1 mice show a tail deformity, and F2 mice did not show any tail deformity. **(C,D)** The body weight and tail length of male F1 mice were lower than those of male control mice from postnatal week 3–week 5 [two-way analysis of variance (ANOVA) followed by Bonferroni *post-hoc* test, *n* (CTRL vs. F1) = 14:14, ***p < 0.001]. **(E,F)** Compared to CTRL group mice, male F2-Con (without prenatal baclofen intervention) and male F2-Int (with prenatal baclofen intervention) mice did not show a significant difference in body weight, but the tail length of F2-Con and F2-Int mice was lower than that of male CTRL group mice in postnatal week 3–week 5 [two-way analysis of variance (ANOVA) followed by Bonferroni *post-hoc* test, *n* (CTRL vs. F2-Con vs. F2-Int) = 14:12:13, ***p < 0.001]. All data for all figures are plotted as the mean ± SEM. **(G)** A representative timeline of the experimental process. E: embryonic day; P: postnatal day; F0: CTRL mice; F1: first-generation mice (VPA-exposed mice); F2: second-generation mice (offspring of VPA-exposed mice); F1-Con: F1 mice with no oral baclofen treatment; F1-Int: F1 mice with oral baclofen treatment; F2-Con: F2 mice with no prenatal baclofen treatment; F2-Int: F2 mice with prenatal baclofen treatment; **#** indicates a comparison between the CTRL group and F2-Con groups; * indicates a comparison between the control (CTRL) group and F2-Int groups.

The pregnant mice (F0 mice) were housed separately and divided into vehicle- and VPA-exposed groups. VPA (Sigma Aldrich, St. Louis, MO, USA) was purchased and dissolved in 0.9% saline at a 10 mg/ml concentration. Prenatal VPA exposure was induced using a new method (49) in which female mice in the VPA-exposed group received two doses each of 300 mg/kg VPA on embryonic days 10 (E10) and E12, and vehicle-exposed group females were injected with the same amount of physiological saline (NS) on the same days. The female mice raised their litters. Male offsprings (F1 mice) of VPA-exposed group females and male offsprings (CTRL mice) of vehicle-exposed group females were weaned on postnatal day 21 (P21) and labeled with ear tags. Because the ASD-related behavior of male VPA-exposed mice is more stable than that of female mice in this model (10, 44, 50), male offspring were used in all subsequent experiments (Figure 1A). Considering the differences in characteristics between different breeding pairs, we equally divided the litters of VPA model mice into a baclofen intervention group and a control group, excluding the last mouse when the number of males in a litter was odd. We also ensured that the fathers of the CTRL and F1 mice were the same male mice to the greatest extent possible to reduce the “litter effect” (51).

#### Breeding of F2 Mice

Breeding pairs of F1 mice with no oral baclofen treatment were obtained from the same litter and were ~10 weeks of age. Female mice weighing 20–25 g and male mice weighing 22–25 g were used. The breeding process was the same as that mentioned above (Figure 1A), and male offspring (F2 mice) were used in all subsequent experiments.

### Drug Administration

#### Baclofen Administration to F1 Mice

Baclofen (MedChemExpress, New Jersey, USA) was administered to male F1-Int group mice (F1 mice with oral baclofen treatment) through the drinking water at a dose of 0.5 mg/ml, and CTRL group mice and F1-Con group mice (F1 mice with no oral baclofen treatment) were provided normal drinking water (control water) from weaning (P21) until the end of the experiment (P60) (Figure 1G). The baclofen dose was selected based on the effective dose for neuropathy and ASD-like symptoms in experimental animal studies (26, 30, 52, 53). We implemented many measures to ensure that each animal received the exact dose of baclofen. These measures include ensuring a constant temperature and humidity in the room, changing drinking water containing drugs daily, placing mice of approximately the same weight in the same cage, and restricting the number of breeding animals in the cage (not more than 4 mice per cage).

Aspartame (MedChemExpress, New Jersey, USA) was added at a concentration of 0.1% to reduce the bitter taste of baclofen. Control water contained aspartame only. All drinking water was refreshed three times a week.

#### Baclofen Administration to F2 Mice

Baclofen was administered through the drinking water using the method described above 3 days before mating to ensure that a stable baclofen concentration was achieved in breeding pairs. The pregnant F1 mice were administered baclofen until delivery. Control pregnant females received normal drinking water during the same period (Figure 1G).

### Growth and Development

The body weight and tail length of all offspring mice (F1 and F2 mice with no oral baclofen treatment) were recorded at weeks 3, 4, and 5 after birth. Pup weight was measured by placing the mouse on the balance and obtaining the reading after the mouse had remained still for 2 s. Pups were weighed twice, and the mean value was calculated. The pup tail length measurement started from the root of the tail, and the length of the tail was measured in the straight state.

A crooked tail was observed in mice and recorded at week 2 after birth (Figure 1B).

### Autism-Related Behavioral Tests

The social interaction test and marble-burying test were completed with F1 and F2 mice in the 8th week after birth, the novel object recognition task was completed in the 7th week after birth, and the open-field task and open-field habituation task were completed in the 7th week after birth. All behaviors of the animals were recorded using a computerized video tracking system (SMART 3.0, Panlab, Spain).

#### Social Interaction Test

The mice were tested in an automated three-chambered social approach apparatus as previously described (27, 28). The test had two testing phases: (A) the sociability phase (scene 1), in which an unfamiliar mouse (stranger 1) was placed inside a plastic cage in one of the side chambers, an empty cage was placed in the other chamber, and the test mouse was allowed to freely explore the apparatus for 5 min; and (B) the preference for social novelty phase (scene 2), in which a second unfamiliar mouse (stranger 2) was placed inside the cage in the opposite chamber, and the test mouse was allowed to freely explore the apparatus for 5 min. The total time spent in each region and the time spent sniffing the stranger mouse and the empty cage were recorded. The social preference index (SPI) was calculated as (stranger 2 time)/(stranger 1 time + stranger 2 time) in scene 2. The tasks started at ~9:30 a.m.

#### Novel Object Recognition Task

The novel object recognition task was performed using a previously reported method with slight modifications (30, 54). The animals were placed in a box containing two identical objects and allowed to explore for 5 min (scene 1). After an interval of 30 s, one object was replaced with a novel object, and the animals were allowed to explore the objects for 5 min (scene 2). The discrimination index (DI) was calculated as (novel object time)/(novel object time + familiar object time). The tasks started at ~9:30 a.m.

#### Open-Field Task and Open-Field Habituation Task

The open-field boxes were made of wood (50.0 × 50.0 × 40.0 cm), and an overhead camera was used for automatic tracking of animal behaviors using SMART 3.0 software. The box was divided into two zones: an “inner” zone (a 30 × 30 cm^2^ central square) and an “outer” zone (10 cm from the walls). The duration of the test was 10 min (task 1).

Inspired by a previous report (30), we repeated the open-field test at 24-h intervals and measured the same indexes (task 2). The open-field exploration index (OFEI) was calculated as the distance traveled in the inner zone/the total distance traveled. The tasks started at ~9:30 a.m.

#### Marble-Burying Test

The tested mouse was placed in a black cage containing 16 marbles arranged in a 4 × 4 grid on clean rice husk bedding up to 5 cm in height. Before the test began, each mouse was acclimated to the cage with rice husk bedding without marbles for 3 min of habituation. The duration of the test was 10 min. Marbles with >75% of their surface buried in the bedding were counted and recorded. Digital images and movies of the marbles were captured during the test period. The numbers of buried marbles and actions (strong and obvious digging or burial movement) were counted from the digital images and movies by trained persons (*n* = 3) who were not associated with this experiment. The operation of the marble-burying test is relatively simple, does not require much energy from the experimental researchers, and observes the active state of mice at night. We performed the test at ~21:30 at night to save time in the experiment.

### Golgi-Cox Staining

In this study, we used the Golgi-Cox staining method to observe the dendritic spines of cerebral neurons. Golgi staining is a powerful technique for providing a complete, detailed representation of a single neuron. With this staining procedure, neuronal spines are observed, which are located on dendrites, receive electric signals from other neurons and are involved in neuronal plasticity. After deep anesthesia was induced with isoflurane, the mice were decapitated, and the brains were removed in a low-temperature environment (operating on ice) and soaked in a mixed AB liquid (FD Rapid GolgiStain™ Kit, NeuroTechnologies, Ellicott City, MD, USA). After 3 weeks, brain tissues were sliced with a vibrating slicer (VT1000S; Leica, Germany) and soaked in liquid C. The thickness of each slice was 100 μm. Five days later, slices were stained with dye solution (solution D:solution E:distilled water; 1:1:2) for 10 min, after which the slices were rinsed with distilled water, dehydrated with an ascending series of ethanol solutions, and cleared in xylene for more than 2 h. Finally, the slices were sealed on slides with neutral resin and dried in the dark.

### Dendritic Spine Analysis

Images of spines in selected brain regions (mPFC; ventral and dorsal HC) were obtained with the Extended Depth of Focus module of a Nikon Eclipse microscope (Shanghai, China), 3D dendritic spine images were combined into a plan view, and ImageJ (Fiji) analysis software was used to evaluate the dendritic spine density in the images.

### Statistical Analysis

Statistical analyses were performed using GraphPad Prism 8.0 software. The results of the statistical tests were considered significant at ^*^p < 0.05, ^**^p < 0.01, and ^***^p < 0.001. Data are presented as the means ± SEM. The body weight and tail length of mice recorded weekly were analyzed using two-way ANOVA. The data from the social interaction test, novel object recognition task and the spine morphological study were analyzed using one-way ANOVA. Open-field task (task 1) and open-field habituation task (task 2) were analysed using paired Student's *t*-test for comparing the differences between different tasks in the same group and one-way ANOVA for comparing the differences among groups in the same task. Marble burying test data were analysed using Pearson correlation, linear regression analysis and one-way ANOVA. All ANOVAs were followed by Bonferroni *post hoc* test to compare the differences among the groups.

## Results

### Tail Malformations in F1 Mice but Not in F2 Mice

Neural tube defects (NTDs) may result from genetic mutations, malnutrition or exposure to teratogens during gestation (55). Tail malformations are often used as a sign of successful modeling in F1 mice (11, 56). The crooked tail phenotype was observed in all F1 mice but was not observed in all F2 mice in the present experiment (Figure 1B), indicating that the crooked tail phenotype associated with NTDs caused by VPA exposure in F1 mice was not transmitted to F2 mice.

### Growth Retardation in F1 Mice

We measured the body weight and tail length of male pups weekly between postnatal days 14 and 35 (Figures 1C,D). The body weight of F1 mice was significantly lower than that of control (CTRL) mice in weeks 3–5 (*F*_1,78_ = 67.47, week 3: ^***^p < 0.0001; week 4: ^***^p < 0.0004; week 5: ^***^p < 0.0001). The tail length of F1 mice was also significantly shorter than that of control mice in weeks 3–5 (*F*_1,78_ = 255.0, week 3: ^***^p < 0.0001; week 4: ^***^p < 0.0001; week 5: ^***^p < 0.0001). The results indicated that F1 mice exhibited severe postnatal growth retardation. This finding is consistent with the characteristics of developmental delay in VPA model mice.

### Normal Body Weight Growth but Tail Dysplasia in F2 Mice

The body weights of F2-Con (offspring of F1 mice without prenatal baclofen intervention) mice and F2-Int (offspring of F1 mice with prenatal baclofen intervention) mice were not significantly different from those of male CTRL mice in weeks 3–5. However, the tail length of F2-Con and F2-Int pups was significantly shorter than that of CTRL mice in weeks 3–5 (*F*_2,108_ = 143.6, CTRL vs. F2-Con, week 3: ^###^*p* < 0.0001; week 4: ^###^*p* < 0.0001; week 5: ^###^*p* < 0.0001); (*F*_2,108_ = 143.6, CTRL vs. F2-Int, week 3: ^***^p < 0.0001; week 4: ^***^p < 0.0001; week 5: ^***^p < 0.0001). No significant difference was observed in tail length between F2-Con and F2-Int pups (Figures 1E,F). Based on these results, F2 mice exhibited tail dysplasia but did not exhibit an altered body weight. The explanation for this result may be related to the reduced VPA exposure-induced damage in F2 mice compared with F1 mice, but the decrease in tail length in F2 mice may be related to a mild NTD.

### Baclofen Treatment Corrected Sociability Deficits in F1 Mice

During scene 1, “sociability” was defined as the propensity to spend time in the cage containing stranger 1 compared with the time spent alone in the identical but empty opposite cage. The session indicates the interest in social cues of tested mice (6, 57–60).

**Figure 2:**
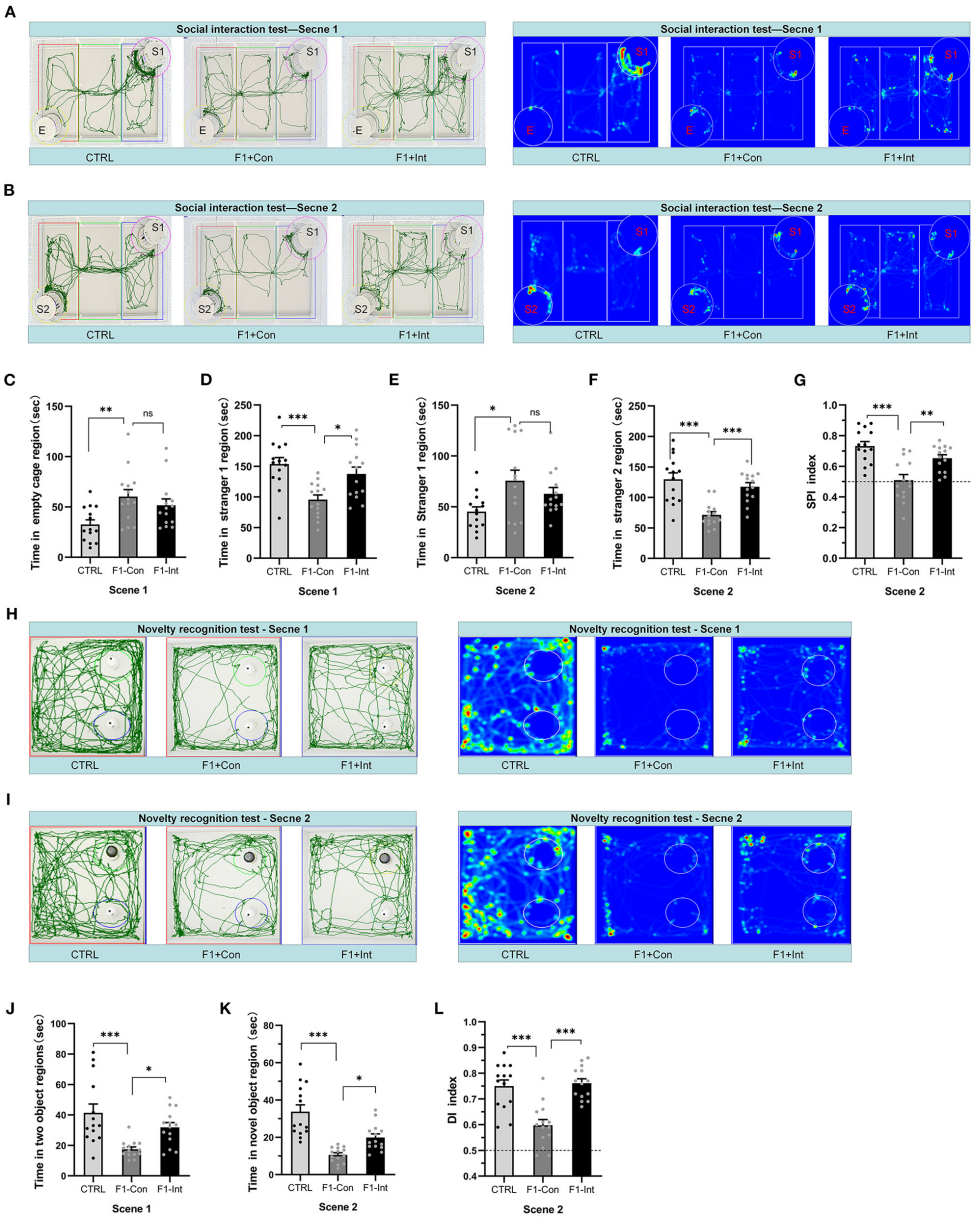
Baclofen treatment corrected social interaction and novelty recognition deficits in F1 mice. S1 = stranger 1 mouse; S2 = stranger 2 mouse; E = empty. **(A)** Representative traces and heatmaps of mice in the sociability phase (scene 1). **(B)** Representative traces and heatmaps of mice in the preference for the social novelty phase (scene 2). **(C)** The time that tested mice entered the region of empty cage for sniffing in scene 1. **(D)** The time that tested mice entered the region containing stranger 1 for sniffing in scene 1. **(E)** The time that tested mice entered the region containing stranger 1 for sniffing in scene 2. **(F)** The time that tested mice entered the region containing stranger 2 for sniffing in scene 2. **(G)** The SPI of tested mice in scene 2. **(H,I)** Representative traces and heatmaps of tested mice in the novel object recognition task. **(H)** Traces of mice exploring the regions containing the two similar objects (white plastic bottles) in phase 1 (scene 1). **(I)** Traces of mice exploring the region containing the novel object (black glass bottle) in phase 2 (scene 2). This test was used to assess novelty recognition ability. **(J)** The total time spent sniffing the two objects by each group of mice in scene 1. **(K)** The time spent sniffing the novel object by each group of mice in scene 2. **(L)** The discrimination index (DI) for each group of mice in scene 2. **(C–G,J–L)** One-way ANOVA followed by the Bonferroni *post-hoc* test was used: *p < 0.05, **p < 0.01, ***p < 0.001. All data for all figures are plotted as the mean ± SEM. Each group had 14 mice (*n* = 14)

As the Figure 2A showed, the F1-Con mice (F1 mice with no oral baclofen treatment) spent more time examining the empty cage than the CTRL mice (*F*_2,39_ = 5.56, ^**^p = 0.0082) in scene 1 (Figure 2C). F1-Con mice spent less time in the region of the cage containing stranger 1 than CTRL mice and F1-Int mice (F1 mice with oral baclofen treatment) (*F*_2,39_ = 9.37, CTRL vs. F1-Con: ^***^p = 0.0005; F1-Con vs. F1-Int: ^*^p = 0.0131, Figure 2D). Therefore, F1-Con mice exhibited obvious sociability deficits, and baclofen treatment corrected the deficits.

### Baclofen Treatment Corrected Deficits in the Preference for Social Novelty in F1 Mice

As the Figure 2B showed, during scene 2, “preference for social novelty” was defined as the propensity to spend time with a new stimulus mouse (stranger 2) rather than with the same stimulus mouse (stranger 1) encountered in scene 1. The session indicates interest in novel social cues of the tested mouse (6, 57–60).

In scene 2, F1-Con mice spent less time in the chamber containing stranger 2 than CTRL/F1-Int mice (*F*_2,39_ = 15.58, CTRL vs. F1-Con:^***^p < 0.0001; F1-Con vs. F1-Int:^***^p = 0.0005, Figure 2F); F1-Con mice spent more time in the chamber containing stranger 1 than CTRL mice (*F*_2,39_ = 4.26, CTRL vs. F1-Con: ^*^p = 0.0179, Figure 2E). More importantly, the SPI of CTRL/F1-Int mice was significantly higher than that of F1-Con mice (*F*_2,39_ = 14.14, CTRL vs. F1-Con: ^***^p < 0.0001; F1-Con vs. F1-Int: ^**^p = 0.005, Figure 2G). The results revealed that the F1-Con mice exhibited an obvious deficit in the preference for social novelty and that baclofen corrected the deficits.

### Baclofen Treatment Corrected Novelty Recognition Deficits in F1 Mice

As the Figures 2H,I showed, similar to previous reports using other ASD mouse models (28, 30), F1-Con mice showed deficits in the preference for novel objects compared with CTRL mice (CTRL vs. F1-Con: *F*_2,39_ = 9.48, ^***^p = 0.0003, scene 1, Figure 2J; *F*_2,39_ = 22.22, ^***^p < 0.0001, scene 2, Figure 2K). Baclofen treatment increased the amount of time F1 mice spent in the region containing the novel object (F1-Con vs. F1-Int: *F*_2,39_ = 9.48, ^*^p = 0.0395, scene 1, Figure 2J; *F*_2,39_ = 22.22, ^*^p = 0.0339, scene 2, Figure 2K). The DI is a valuable index that reflects object recognition memory and the preference for novel objects. The DI of F1 mice without baclofen treated, was lower than that of CTRL mice (*F*_2,39_ = 18.51, CTRL vs. F1-Con: ^***^p < 0.0001, scene 2, Figure 2L) and baclofen-treated F1 mice (F1-Con vs. F1-Int: *F*_2,39_= 18.51, ^***^p < 0.0001, scene 2, Figure 2L). Thus, baclofen treatment corrected the deficits in the preference for novel objects in VPA-exposed mice.

### Baclofen Treatment Corrected Locomotor and Exploratory Activity Deficits in F1 Mice

**Figure 3:**
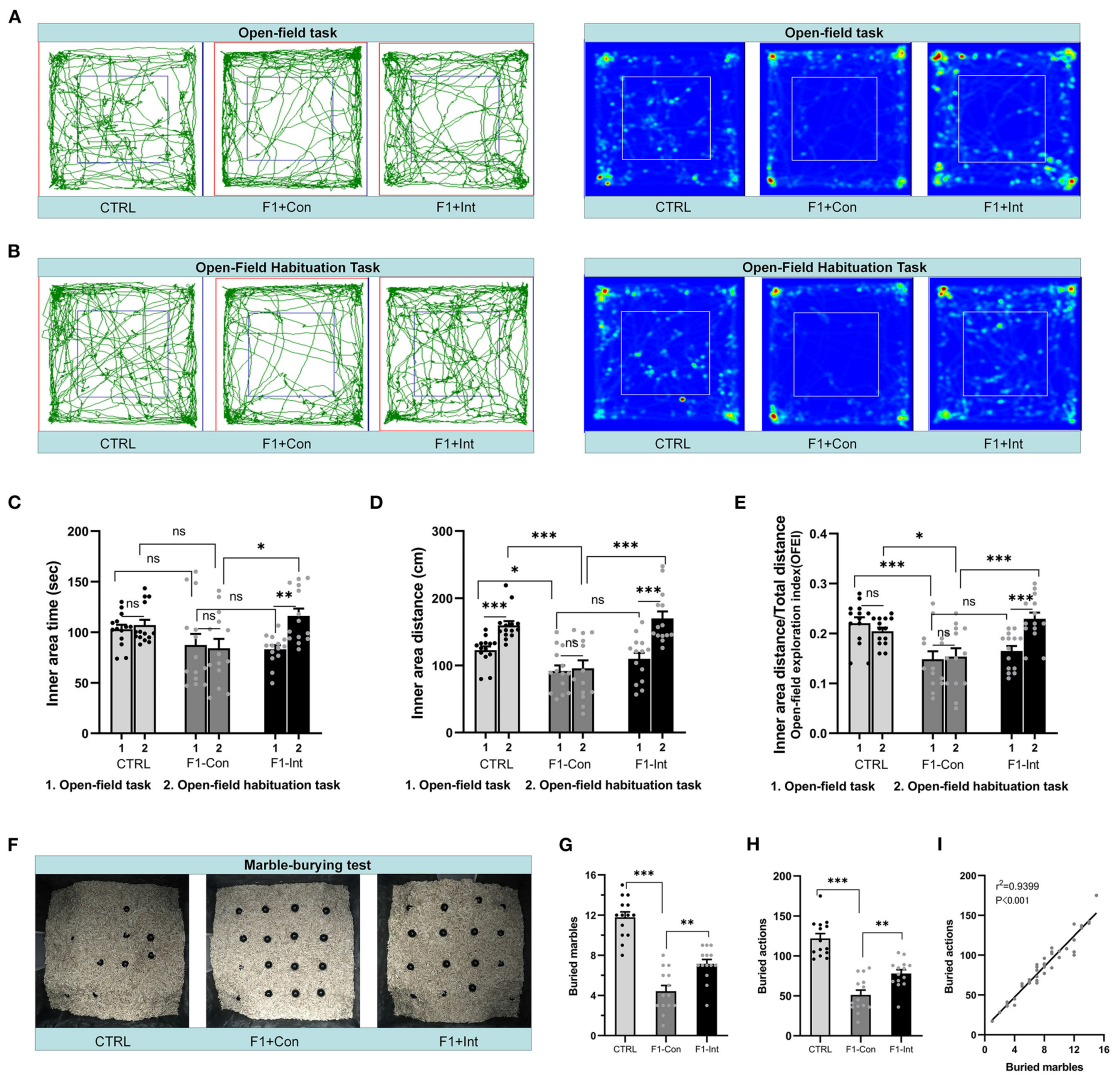
Baclofen treatment corrected the locomotor, exploratory activity and marble-burying deficits in F1 mice. **(A,B)** Representative traces and heatmaps of mice in the open-field task (task 1) and open-field habituation task (task 2). **(C)** Time traveled in the inner zone by mice in the two open-field tasks. **(D)** Distance traveled by mice in the inner zone in the two open-field tests. **(E)** The open-field exploration index (OFEI = distance traveled in the inner zone/the total distance traveled) of mice in the two open-field tasks. In (**C–E)**, Student's paired *t*-test was used to compare the differences in behavior in the two tasks between each group of mice, and one-way ANOVA followed by the Bonferroni *post-hoc* test was used to compare the difference in the same task across the four groups; *p < 0.05, **p < 0.01 ***p < 0.001. All data for all figures are plotted as the mean ± SEM. Each group had 14 mice (*n* = 14). **(F)** Representative marble-burying maps after the marble-burying test. **(G)** The numbers of buried marbles for each group of mice. **(H)** The number of burying actions for each group of mice. In **(G,H)**, one-way ANOVA followed by the Bonferroni *post-hoc* test was used to compare the differences among groups; **p < 0.01, ***p < 0.001. All data for all figures are plotted as the mean ± SEM. Each group had 14 mice (*n* = 14). **(I)** Pearson correlation and linear regression analysis between the number of buried marbles and the number of burying actions. All mice were included (*n* = 42) in the analysis, and there was a linear correlation between the number of buried marbles and the number of burying actions.

As the Figure 3A showed, in the open-field task (task 1), F1-Con mice showed lower locomotor and exploratory behaviors than CTRL mice, including decreases in the distance traveled in the inner area (CTRL vs. F1-Con: *F*_2,39_ = 4.095, ^*^p = 0.0210, Figure 3D) and the OFEI (CTRL vs. F1-Con: *F*_2,39_ = 8.992, ^***^p = 0.0007, Figure 3E). However, no significant differences in the time traveled in the inner area were observed compared with CTRL mice (CTRL vs. F1-Con: *F*_2,39_ = 2.118, ^ns^*p* = 0.3932, Figure 3C). Baclofen treatment did not increase the indicators of locomotor/exploratory activity in F1 mice in task 1 (F1-Con vs. F1-Int: *F*_2,39_ = 2.118, ^ns^*p* > 0.9999, Figure 3C; *F*_2,39_ = 4.095, ^ns^*p* = 0.3090, Figure 3D; *F*_2,39_ = 8.992, ^ns^*p* > 0.9999, Figure 3E). Based on these results, VPA model mice exhibited deficits in locomotor and exploratory activity in a new open environment, and treatment with baclofen did not exert a positive effect on ameliorating these changes in locomotor and exploratory behaviors in F1 mice in task 1.

Inspired by a previous report (30), we redesigned the open-field habituation task (task 2; the two tasks were performed at a 24 h interval) to further evaluate the therapeutic effects of baclofen. As the Figure 3B showed, in contrast to task 1, CTRL mice and F1-Int mice traveled a greater distance in the inner area in task 2 (CTRL: *t* = 5.612, df = 13, ^***^p < 0.0001; F1-Int: *t* = 6.114, df = 13, ^***^p < 0.0001, Figure 3D), but not VPA-exposed mice (F1-Con: *t* = 0.3125, df = 13, ^ns^*p* = 0.7596, Figure 3D). In addition, in contrast to task 1, the OFEI of F1-Int mice was also increased in task 2 (F1-Int: *t* = 6.172, df = 13, ^***^p < 0.0001, Figure 3E), but not in F1-Con mice (F1-Con: *t* = 0.2737, df = 13, ^ns^*p* = 0.7886, Figure 3E).

In addition, the indicators of the distance traveled in the inner area or the OFEI of F1-Con mice were lower than those of CTRL mice (CTRL vs. F1-Con: *F*_2,39_ = 16.77, ^***^p = 0.0001, Figure 3D; *F*_2,39_ = 9.218, ^*^p = 0.0224, Figure 3E) and F1-Int mice (F1-Con vs. F1-Int: *F*_2,39_ = 16.77, ^***^p < 0.0001, Figure 3D; *F*_2,39_ = 9.218, ^***^p = 0.0004, Figure 3E) in task 2. Baclofen treatment also increased the time traveled in the inner area by F1 mice in task 2 (F1-Con vs. F1-Int: *F*_2,39_ = 4.997, ^*^p = 0.0119, Figure 3C). The results revealed that baclofen treatment corrected locomotor and exploratory activity deficits in F1 mice in an open environment that they had been habituated to 24 h previously.

In conclusion, we propose that the differences in the results of the two tasks may have been related to decreases in anxiety and fear. This anxiolytic effect may have been caused by previous experience exploring the same apparatus and familiarity with the environment. However, treatment with baclofen substantially corrected the deficits in F1 mice in recognizing a familiar environment in the open-field habituation task.

### Linear Correlation Between the Number of Buried Marbles and Burying Actions in the Marble-Burying Test

We counted the number of marbles buried and burying actions of all groups of mice in the marble-burying test and found that these two measures were linearly correlated (*r*^2^ = 0.9399, ^***^p < 0.001/Y = 9.628^*^X + 8.733) (Figure 3I). This showed that the marble burying actions in the test were effective, and the time of test is appropriate.

### Baclofen Treatment Corrected Marble-Burying Deficits in F1 Mice

As the Figure 3F showed, the numbers of buried marbles and burying actions were lower in F1 mice than in CTRL mice (CTRL vs. F1-Con: *F*_2,39_ = 51.94, ^***^p < 0.0001, Figure 3G; *F*_2,39_ = 40.452, ^***^p < 0.0001, Figure 3H), indicating that F1 mice showed marble-burying deficits. Baclofen treatment increased these parameters in F1 mice (F1-Con vs. F1-Int: *F*_2,39_ = 51.94, ^**^p = 0.0019, Figure 3G; *F*_2,39_ = 40.452, ^**^p = 0.005, Figure 3H), indicating that baclofen exerted a therapeutic effect on marble-burying deficits in F1 mice.

### Baclofen Treatment Corrected Sociability Deficits in F2 Mice

**Figure 4:**
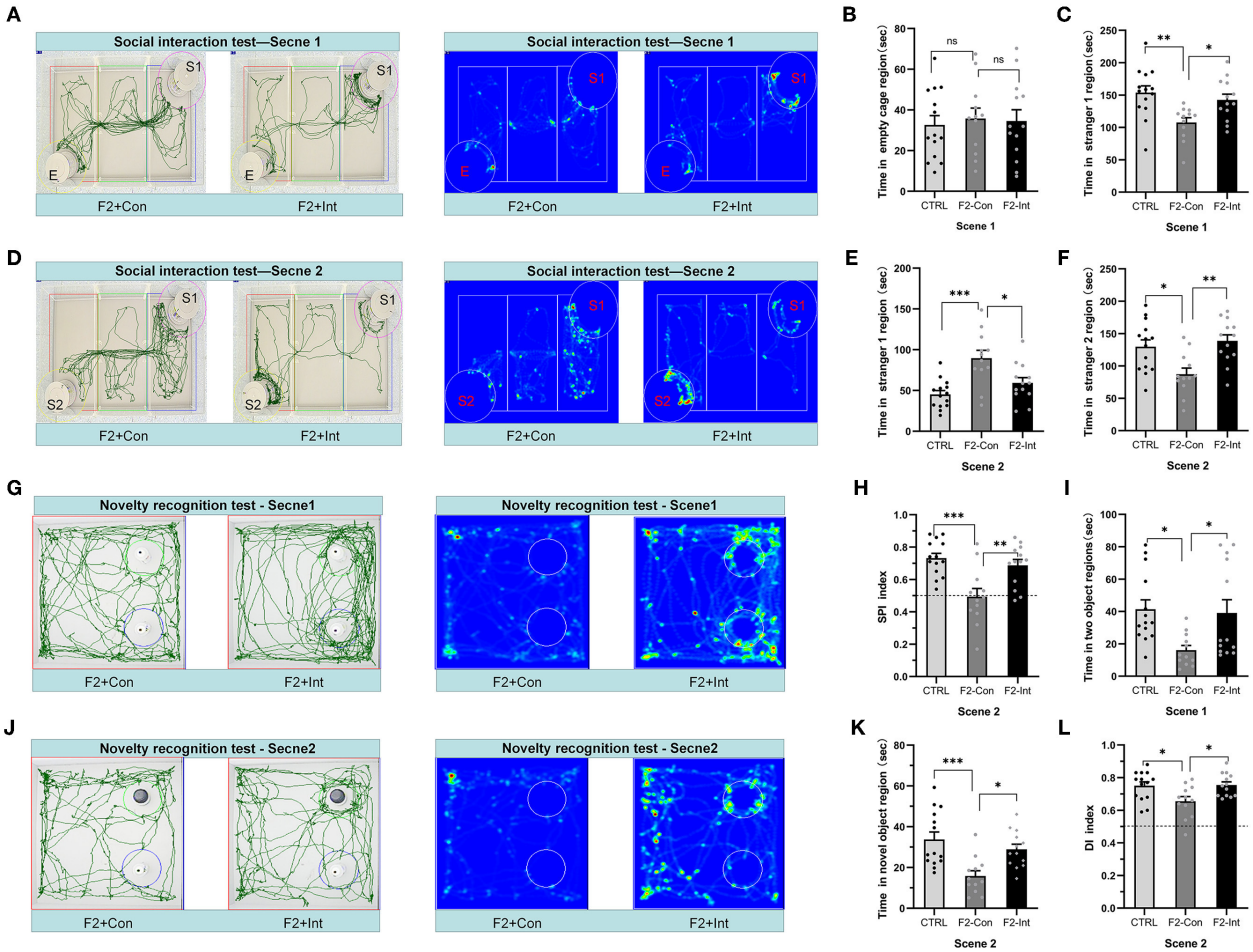
Prenatal baclofen administration corrected social interaction deficits and novelty recognition deficits in F2 mice in the social interaction test. **(A,D)** In the social interaction test, representative traces and heatmaps from tested mice in the sociability phase (scene 1) and preference for social novelty phase (scene 2). **(B)** The time that tested mice entered the region of empty cage for sniffing in scene 1 of the social interaction test. **(C)** The time that tested mice entered the region containing stranger 1 for sniffing in scene 1 of the social interaction test. **(E)** The time that tested mice entered the region containing stranger 1 for sniffing in scene 2 of the social interaction test. **(F)** The time that tested mice entered the region containing stranger 2 for sniffing in scene 2 of the social interaction test. **(G,J)** Representative traces of tested mice in the novel object recognition task. **(G)** Traces and heatmaps of mice exploring the regions containing the two similar objects (white plastic bottles) in phase 1 (scene 1). **(H)** The SPI of tested mice in scene 2 of the social interaction test. **(I)** The total time spent sniffing the two objects by each group of mice in scene 1 of the novel object recognition task. **(J)** Traces and heatmaps of mice exploring the region containing the novel object (black glass bottle) in phase 2 (scene 2) of the novel object recognition task. This test was used to assess novelty recognition ability. **(K)** The time spent sniffing the novel object by each group of mice in scene 2 of the novel object recognition task. **(L)** The discrimination index (DI) for each group of mice in scene 2 of the novel object recognition task. **(B,C,E,F,H,I,K,L)** One-way ANOVA followed by the Bonferroni *post hoc* test was used to compare the differences among groups: *p < 0.05, **p < 0.01, ***p < 0.001. All data for all figures are plotted as the mean ± SEM. n (CTRL: F2-Con: F2-Int) = 14:12:13.

As the Figure 4A showed, the results did not reveal a significant difference in time spent in the empty cage in scene 1 between the three groups of mice (Figure 4B). F2-Con mice spent less time in the region of the cage containing stranger 1 than the CTRL mice and F2-Int mice (*F*_2,36_ = 6.722, CTRL vs. F1-Con: ^**^p = 0.0032; F2-Con vs. F2-Int: ^*^p = 0.0362, Figure 4C).

Based on these results, the F2-Con mice exhibited obvious sociability deficits, and baclofen treatment corrected the deficits.

### Baclofen Treatment Corrected Deficits in the Preference for Social Novelty in F2 Mice

As the Figure 4D showed, in scene 2, F2-Con mice spent less time in the chamber containing stranger 2 than the CTRL/F2-Int mice (*F*_2,36_ = 7.465, CTRL vs. F2-Con: ^*^p = 0.0128; F2-Con vs. F2-Int: ^**^p = 0.0027, Figure 4F); F2-Con mice spent more time in the chamber containing stranger 1 than the CTRL mice and F2-Int mice (*F*_2,36_ = 10.46, CTRL vs. F2-Con: ^***^p = 0.0002; F2-Con vs. F2-Int: ^*^p = 0.0136 Figure 4E). More importantly, the SPI of CTRL/F2-Int mice was significantly higher than that of F2-Con mice (*F*_2,36_ = 10.42, CTRL vs. F2-Con: ^***^p = 0.0003; F1-Con vs. F2-Int: ^**^p = 0.004, Figure 4H). The results revealed that the F2-Con mice exhibited an obvious deficit in the preference for social novelty and that baclofen corrected the deficits.

### Prenatal Baclofen Treatment Corrected Novelty Recognition Deficits in F2 Mice

As the Figures 4G,J showed, F2-Con mice showed deficits in the preference for novel objects compared with CTRL mice (CTRL vs. F2-Con: *F*_2,36_ = 5.020, ^*^p = 0.0184, scene 1, Figure 4I; *F*_2,36_ = 9.076, ^***^p = 0.0006, scene 2, Figure 4K; *F*_2,36_ = 5.175, ^*^p = 0.0264, scene 2, Figure 4L). Prenatal baclofen treatment increased the DI and the amount of time F2 male mice spent in the region containing the novel object (CTRL vs. F2-Con: *F*_2,36_ = 5.020, ^*^p = 0.0401, scene 1, Figure 4I; *F*_2,36_ = 9.076, ^*^p = 0.0153, scene 2, Figure 4K; *F*_2,36_ = 5.175, ^*^p = 0.0213, scene 2, Figure 4L). Prenatal baclofen treatment corrected deficits in the novel object preference of F2 mice.

### Prenatal Baclofen Treatment Corrected Locomotor and Exploratory Activity Deficits in F2 Mice

**Figure 5:**
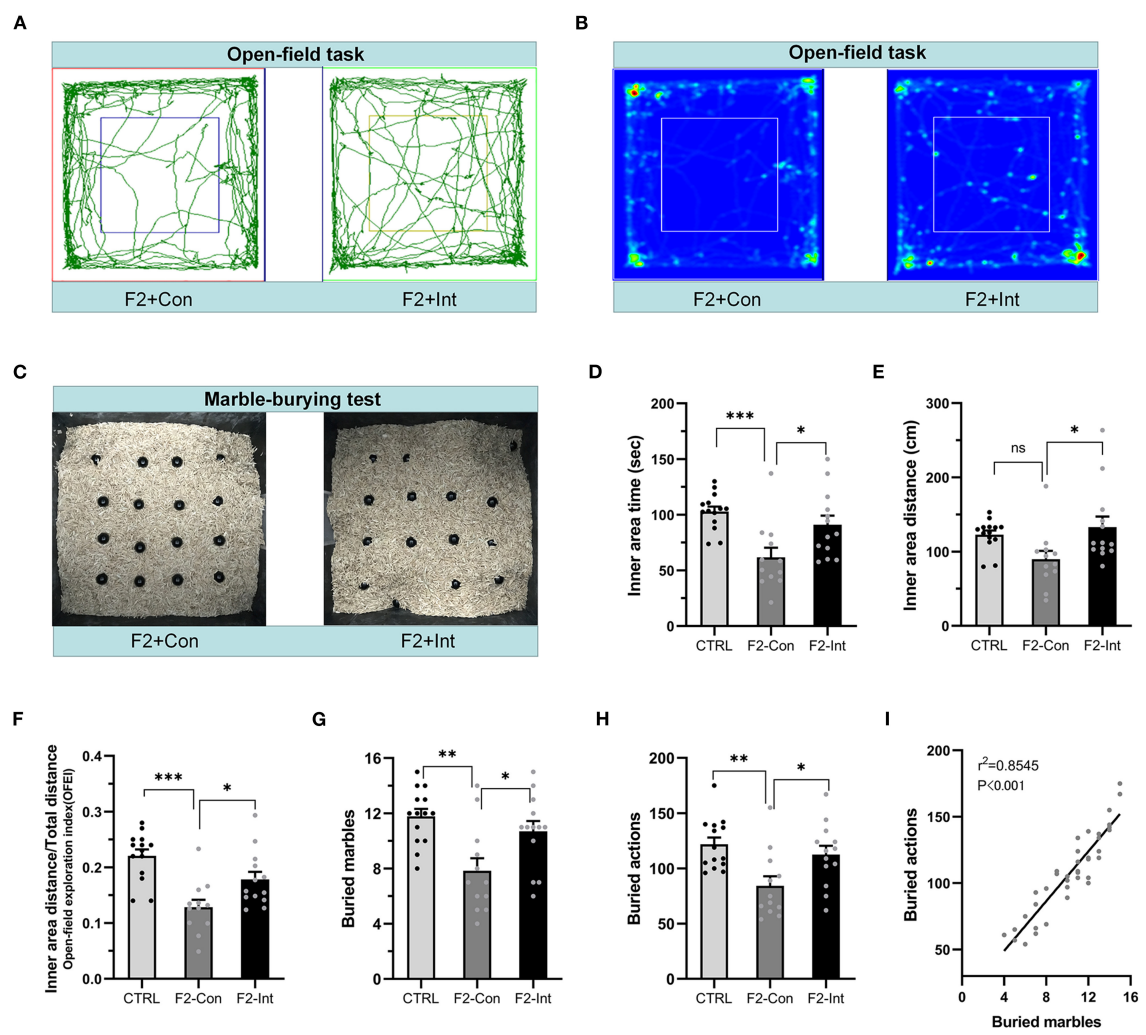
Prenatal baclofen administration corrected locomotor, exploratory activity, and marble-burying deficits in F2 mice. **(A)** Representative traces of mice in the open-field task. **(B)** Representative heatmaps of traces of mice corresponding to **(A)**. **(C)** Representative marble-burying maps after the marble-burying test. **(D)** Time traveled in the inner zone by mice. **(E)** Distance traveled in the inner zone by mice. **(F)** The open-field exploration index (OFEI = distance traveled in the inner zone/the total distance traveled) of mice. **(G)** The numbers of buried marbles of mice. **(H)** The number of burying actions of mice. In **(D–H)** and **(G–I)**, one-way ANOVA followed by the Bonferroni *post-hoc* test was used to compare the differences among groups: *p < 0.05, **p < 0.01 ***p < 0.001. All data for all figures are plotted as the mean ± SEM. n (CTRL: F2-Con: F2-Int) = 14:12:13. **(I)** Pearson correlation and linear regression analysis between the number of buried marbles and the number of burying actions. All mice were included (*n* = 39) in the analysis, and there was a linear correlation between the number of buried marbles and the number of burying actions.

As the Figures 5A,B showed, in the open-field task, F2-Con mice showed lower locomotor and exploratory behavior than CTRL mice, including decreases in the time traveled in the inner area (CTRL vs. F2-Con: *F*_2,36_ = 8.584, ^***^p = 0.0007, Figure 5D) and in the OFEI (CTRL vs. F2-Con: *F*_2,36_ = 12.650, ^***^p < 0.0001, Figure 5F). F2-Int mice showed greater exploratory and locomotor behaviors than F2-Con mice, including increases in the time traveled in the inner area (F2-Con vs. F2-Int: *F*_2,36_ = 8.584, ^*^p = 0.025, Figure 5D), the distance traveled in the inner area (F2-Con vs. F2-Int: *F*_2,36_ = 4.193, ^*^p = 0.026, Figure 5E), and the OFEI (F2-Con vs. F2-Int: *F*_2,36_ = 12.650, ^*^p = 0.0343, Figure 5F). Thus, F2 male mice exhibited lower levels of locomotor and exploratory behaviors than CTRL mice, and prenatal treatment with baclofen exerted a positive effect on ameliorating locomotor or exploratory behavioral deficits in F2 mice.

### Prenatal Baclofen Treatment Corrected Marble-Burying Deficits in F2 Mice

As the Figure 5C showed, the numbers of buried marbles and burying actions were lower in F2-Con mice than in CTRL mice (CTRL vs. F2-Con: *F*_2,36_ = 7.638, ^**^p = 0.0015, Figure 5G; *F*_2,36_ = 6.548, ^**^p = 0.0037, Figure 5H), indicating that F2 male mice exhibited marble-burying deficits. Baclofen treatment increased these parameters in F2 mice (F2-Con vs. F2-Int: *F*_2,36_ = 7.638, ^*^p = 0.0302, Figure 5G; *F*_2,36_ = 6.548, ^*^p = 0.0401, Figure 5H), indicating that prenatal baclofen treatment exerted a certain therapeutic effect on the marble-burying deficits in F2 mice.

The marbles buried and the burying actions of all groups of mice (CTRL, F2-Con, F2-Int) were linearly correlated in the marble-burying test (*r*^2^ = 0.8545, ^***^p < 0.001/Y = 9.372^*^X + 11.49) (Figure 5I).

### Prenatal Baclofen Treatment Corrected Alterations in Dendritic Spine Density on CA1 Pyramidal Neurons in the HC of F2 Mice

Because dendritic spines play critical roles in synaptic plasticity, we sought to determine whether F2 mice exhibited changes in the total spine density and mature spine density on CA1 pyramidal neurons in the HC. Because the functions of the ventral HC (related to stress, emotion, and affect) and dorsal HC are different (related to cognitive functions) (61), we measured the densities of total dendritic spines and mature dendritic spines on basal and apical dendrites of vertebral neurons in the ventral HC and dorsal HC in the CA1 region.

**Figure 6:**
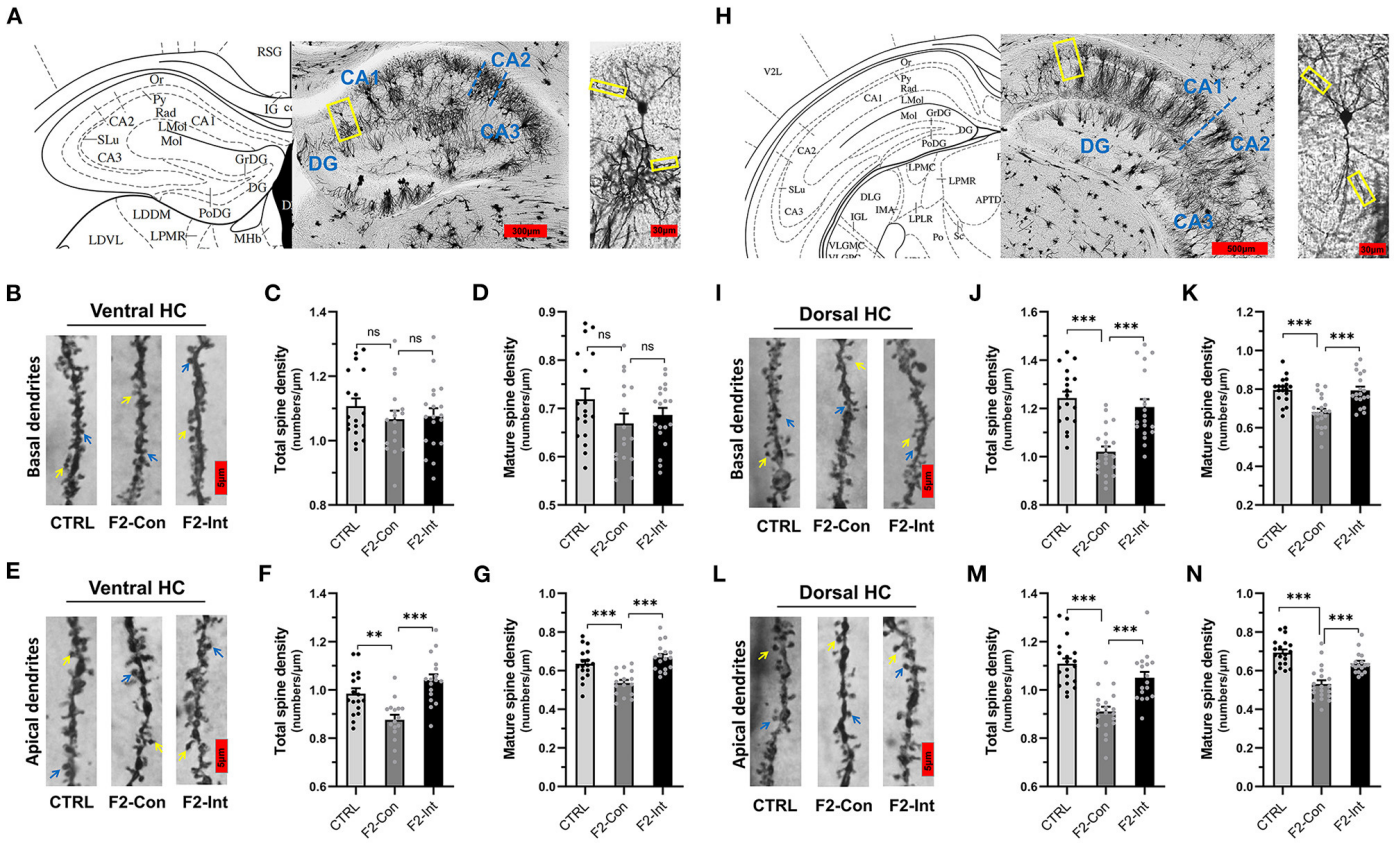
Prenatal baclofen administration corrected the defects in dendritic spines density of CA1 pyramidal neurons in the HC in F2 mice. **(A)** A composite of a representative scanned image of Golgi-Cox-stained slices of the ventral HC and the mouse brain map from *The Mouse Brain in Stereotaxic Coordinates*; scale bar: 300 and 30 μm. **(A/H)** The part inside the yellow rectangle is the neurons and dendrites selected for analysis. The spines were selected from grade 3 basal dendrites and grade 6/7 apical dendrites for analysis. **(B/E)** Representative three-dimensional reconstructed images of the basal/apical dendrites of pyramidal neurons in the CA1 of the ventral HC obtained from CTRL, F2-Con and F2-Int mice. The yellow arrow points to a mushroom spine, and the blue arrow points to a stubby spine. Scale bar: 5 μm. **(C,D)** Summary of spine density on the basal dendrites of CA1 pyramidal neurons in the ventral HC. Mature spines = mushroom spines + stubby spines (CTRL: *n* = 18 dendrites from 3 mice; F2-Con: *n* = 17 dendrites from 3 mice; F2-Int: *n* = 19 dendrites from 3 mice). **(F,G)** Summary of spine density on the apical dendrites of CA1 pyramidal neurons in the ventral HC. Mature spines = mushroom spines + stubby spines (CTRL: *n* = 17 dendrites from 3 mice; F2-Con: *n* = 16 dendrites from 3 mice; F2-Int: *n* = 17 dendrites from 3 mice). **(H)** A composite of a representative scanned image of Golgi-Cox-stained slices of the dorsal HC and the mouse brain map from *The Mouse Brain in Stereotaxic Coordinates*; scale bar: 500 and 30 μm. **(I/L)** Representative three-dimensional reconstructed images of the basal/apical dendrites of pyramidal neurons in the CA1 of the dorsal HC. The yellow arrow points to the mushroom spine, and the blue arrow points to the stubby spine. Scale bar: 5 μm. **(J,K)** Summary of spine density on the basal dendrites of CA1 pyramidal neurons in the HC (CTRL: *n* = 18 dendrites from 3 mice; F2-Con: *n* = 21 dendrites from 3 mice; F2-Int: *n* = 19 dendrites from 3 mice). **(M,N)** Summary of spine density on the apical dendrites of CA1 dorsal neurons in the dorsal HC. Mature spines = mushroom spines + stubby spines (CTRL: *n* = 19 dendrites from 3 mice; F2-Con: *n* =20 dendrites from 3 mice; F2-Int: *n* = 17 dendrites from 3 mice). One-way ANOVA followed by the Bonferroni *post-hoc* test was used to compare the differences among the four groups; *p < 0.05, **p < 0.01, ***p < 0.001. All data for all figures are plotted as the mean ± SEM.

As the Figures 6H,I,L showed, the analysis of mouse brain slices of the dorsal HC with Golgi staining showed that the total spine density and mature spine density (mushroom-shaped + stubby-shaped spines) were significantly lower on pyramidal neurons from F2-Con mice than on those from CTRL mice, including basal dendrites (CTRL vs. F2-Con, total spine density: *F*_2,55_ = 19.52, ^***^p < 0.0001, Figure 6J; mature spine density: *F*_2,55_ = 13.87, ^***^p < 0.0001, Figure 6K) and apical dendrites (CTRL vs. F2-Con, total spine density: *F*_2,53_ = 22.33, ^***^p < 0.0001, Figure 6M; mature spine density: *F*_2,53_ = 24.74, ^***^p < 0.0001, Figure 6N). Our analysis revealed that prenatal baclofen treatment corrected spine density defects in the dorsal HC of F2 mice (F2-Int), including basal dendrites (F2-Con vs. F2-Int, total spine density: *F*_2,55_ = 19.52, ^***^p < 0.0001, Figure 6J; mature spine density: *F*_2,55_ = 13.87, ^***^p = 0.0001, Figure 6K) and apical dendrites (F2-Con vs. F2-Int, total spine density: *F*_2,53_ = 22.33, ^***^p = 0.0001, Figure 6M; mature spine density: *F*_2,53_ = 24.74, ^***^p = 0.0001, Figure 6N).

Similarly, as the Figures 6A,B,E showed, though there were no difference in spine density on basal dendrites of CA1 pyramidal neurons in the ventral HC obtained from three groups (total spine density, *F*_2,51_ = 0.7057, CTRL vs. F2-Con: nsp = 0.8001; F2-Con vs. F2-Int: nsp > 0.9999, Figure 6C) (mature spine density, *F*_2,51_ = 1.709, CTRL vs. F2-Con: nsp = 0.2264; F2- Con vs. F2-Int: nsp > 0.9999, Figure 6D), the analysis showed a lower spine density on apical dendrites of CA1 pyramidal neurons in the ventral HC obtained from F2-Con mice than that obtained from CTRL mice (total spine density: *F*_2,47_ = 13.77, ^**^p = 0.0040, Figure 6F; mature spine density: *F*_2,47_ = 14.98, ^***^p = 0.0008, Figure 6G). Prenatal baclofen treatment corrected the altered spine density on apical dendrites in F2 mice (total spine density: *F*_2,47_ = 13.77, ^***^p < 0.0001, Figure 6F; mature spine density: *F*_2,47_ = 14.98, ^***^p < 0.0001, Figure 6G). In the ventral HC, we did not observe significant differences in the spine density on basal dendrites of CA1 pyramidal neurons among the CTRL, F2-Con, F2-Int groups of mice.

### Prenatal Baclofen Treatment Corrected Defects in Dendritic Spine Density on Pyramidal Neurons in the mPFC of F2 Mice

**Figure 7:**
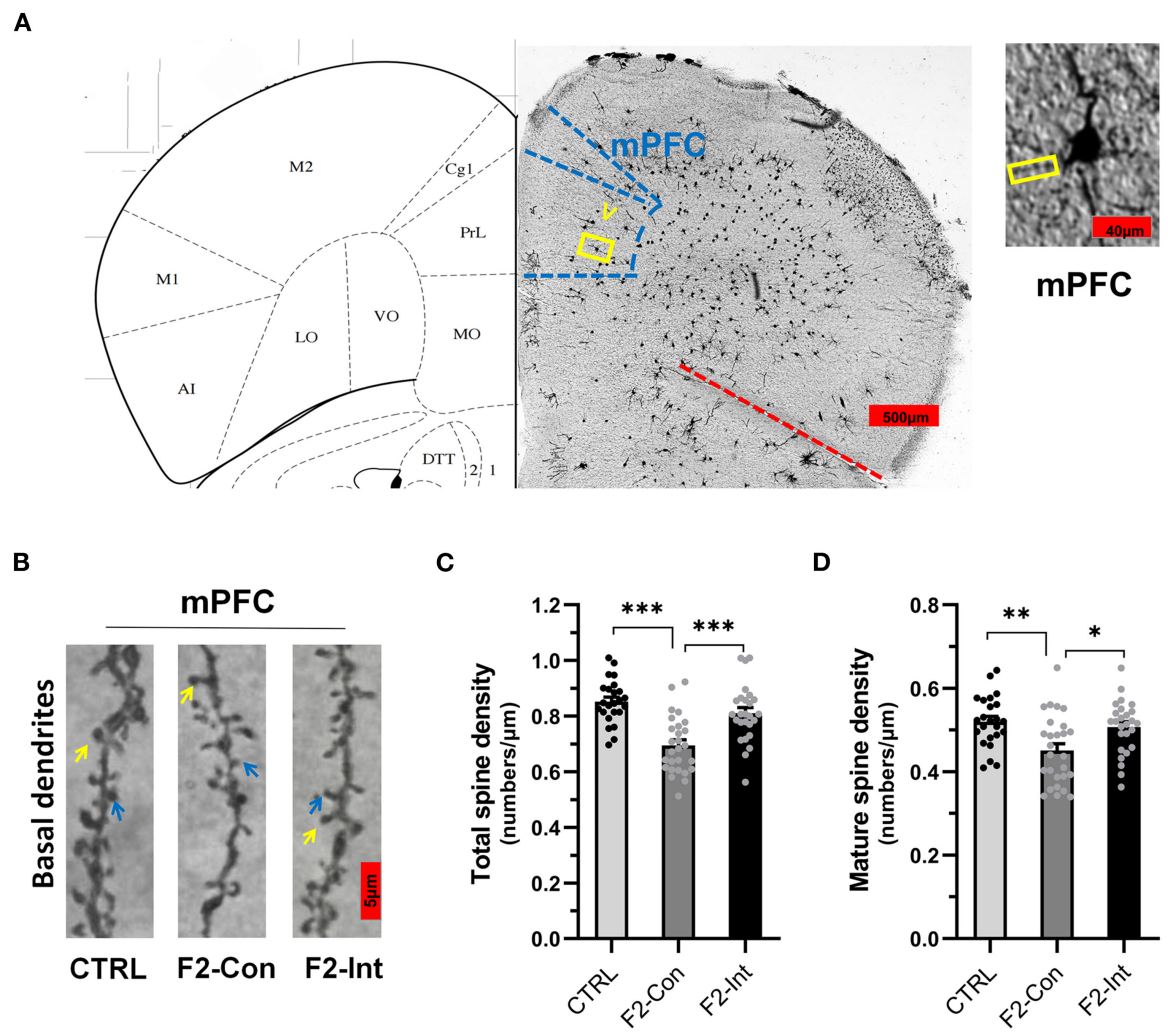
Prenatal baclofen administration corrected the defects in dendritic spines density of pyramidal neurons in the mPFC in F2 mice. **(A)** A composite of a representative scanned image of Golgi-Cox-stained slices of the mPFC and the mouse brain map from *The Mouse Brain in Stereotaxic Coordinates*; scale bar: 500 and 40 μm. **(B)** The part inside the yellow rectangle is the neurons and dendrites selected from layer V in the mPFC for analysis. The spines were selected from grade 3 basal dendrites for analysis. The yellow arrow points to a mushroom spine, and the blue arrow points to a stubby spine. Scale bar: 5 μm. **(C,D)** Summary of spine density on the basal dendrites of mPFC pyramidal neurons. Mature spines = mushroom spines + stubby spines (CTRL: *n* = 24 dendrites from 3 mice; F2-Con: *n* = 27 dendrites from 3 mice; F2-Int: *n* = 26 dendrites from 3 mice). One-way ANOVA followed by the Bonferroni *post-hoc* test was used to compare the differences among the four groups; *p < 0.05, **p < 0.01, ***p < 0.001. All data for all figures are plotted as the mean ± SEM.

As the Figures 7A,B showed, the analysis of mouse mPFC brain slices with Golgi staining showed a lower total spine density and mature spine density on basal dendrites of pyramidal neurons of layer V in F2-Con mice than those in CTRL mice (CTRL vs. F2-Con, total spine density: *F*_2,74_ = 18.79, ^***^p < 0.0001, Figure 7C; mature spine density: *F*_2,74_ = 7.046, ^**^p = 0.0025, Figure 7D). Prenatal baclofen treatment corrected the spine density defects in F2 mice (F2-Con vs. F2-Int, *F*_2,74_ = 18.79, ^***^p = 0.0001, Figure 7C; mature spine density: *F*_2,74_ = 7.046, ^*^p = 0.0151, Figure 7D).

## Discussion

In present preclinical study, we designed a rigorous mouse breeding process and behavioral tests to evaluate the rigor and repeatability of assessments evaluating the efficacy of drug therapy. Baclofen has been used in the clinic for many years as a treatment for spasticity in children and adults with cerebral palsy. Placebo-controlled trials in patients with fragile X syndrome and autism using R-baclofen have shown that the drug is safe and well tolerated in patients with other developmental brain disorders (30). In addition, baclofen crosses the placental barrier. We first designed a long-term oral baclofen experiment for weaned VPA-exposed mice (F1) and found that baclofen administration improved the core autism-like behaviors of F1 mice. Long-term moderate activation of GABAB receptors exerted therapeutic effects on F1 mice during the developmental stage. Subsequent prenatal baclofen treatment of F2 mice showed a similar beneficial effect on ameliorating autism-like behavioral dysfunction. Thus, moderate activation of GABAB receptors during the pregnancy period exerts therapeutic effects on the inheritance of autism-like core behaviors in F2 mice.

Social interaction tests (6, 57–60), novel object recognition tests (30, 54), and open-field tests (11, 30) are classic experiments used to detect autism-like behavior of core symptoms of the ASD diagnosis: social communication and interaction impairments, restricted interests and anxiety-like behaviors. F1 mice that were prenatally exposed to VPA (300 mg/kg) on the gestational day 10 and 12 showed autism-like behaviors, including (1) decreased social interaction parameters; (2) decreased novel object recognition parameters; (3) decreased open-field parameters; and (4) significantly decreased conditioned defense responses in the marble-burying test, as assessed as buried marbles and burying actions. The marble-burying test is commonly used to detect repetitive and stereotypic behavioral indicators. Our experimental results are not consistent with several previous studies (62–64), showing that the numbers of buried marbles and burying actions were lower in F1 mice than in CTRL mice and baclofen exerted a therapeutic effect on marble-burying deficits in F1 mice. This discrepancy may be due to differences in the total test time, acclimation period, test days, standard buried marble, volume and color of the test cage, number of marbles, and other parameters. We suggest that our experimental results provided more support for the marble-burying test as a conditioned defense response (65–67). The behavior of marble burying is more similar to adaptation to a new complicated environment. In addition, our results showed that F2 male mice obviously inherited their parents' core autism-like phenotypes.

The intrinsic pathology of VPA-exposed mice is presumed to provide a model of the environmental/epigenetic origins of epigenetic changes induced by prenatal exposure to VPA. Compared with transgenic models carrying mutations in single autism-associated genes, the model can better reflect many clinical cases of idiopathic autism (11). Neurotransmission regulated by VPA, as a key mechanism, could influence neurodevelopment (11, 68, 69) and regulate gene expression through chromatin remodeling by inhibiting histone deacetylase (HDAC) activity (41, 70). These molecular disturbances have been shown to induce epigenomic disturbances in gametes that may result in abnormal transcription of brain-related genes during fetal and early development, resulting in abnormal neurobehavioral phenotypes in offspring, such as F2 mice (71–74). Furthermore, F2 mice were not only affected by epigenetic information based on changes in histone acetylation but were also exposed to VPA during the gonad development of F1 mice. In other words, F2 mice were also directly exposed to VPA (45). This result may help explain the high degree of inheritance of autism-like behavior in the F2 generation of mice from the F1 mice. Our results showed that after oral treatment with baclofen, the core autism-like behavioral indicators in F1 mice were ameliorated to varying degrees (Figures 2 and 3). Encouraged by the results, we continued to investigate whether prenatal baclofen treatment ameliorated the core autism-like deficits in the F2 generation. The results were similar to those in F1 mice and included the following changes: prenatal baclofen treatment in the F2 generation (1) increased the social time and index, (2) increased the exploration of new objects, (3) increased locomotor and exploratory activities in the open-field test, and (4) increased the number of buried marbles and burying actions.

Synaptic development, maintenance and plasticity under both physiological and pathological conditions are frequently associated with abnormalities in the morphology and numbers of dendritic spines. Disruptions in synaptic plasticity are considered to be the basic neural mechanism underlying various mental diseases. Dynamic changes in dendritic spines play an important role in the formation and refinement of neural circuits and in higher brain neurobehavioral functions. When the density and morphology of dendritic spines change, the structure and function of synapses change accordingly (48). In our previous study, we found that arbaclofen increased the density of basal dendritic spines on neurons in the CA1 region of the dorsal HC in VPA mice (unpublished results). In some studies, the same changes have been reported in F1 mice, and medications can correcte the defect (75–77). Based on these result, we used the Golgi-Cox staining method to study the spine density in the brains of F2 mice, as this parameter is closely related to autism-like behaviors. The spine density was abnormal in the ventral HC, dorsal HC, and mPFC of F2 mice, including reductions in the total spine density and mature spine density. These findings indicated that important brain regions associated with autism in F2 mice exhibited prominent defects in synaptic plasticity and that connectivity was reduced. After prenatal baclofen treatment, this deficiency in F2 mice was reversed.

GABA signaling plays pivotal roles in the initial formation of neuronal networks in the embryonic and early postnatal brain (78), of which GABAB receptors have an extremely important effects in early neural development, involving neuronal survival and migration, developmental pruning, and synaptic formation and maturation (79–81). Previous studies confirmed that mouse models with mutations in GABA receptor subunits showed obvious social deficits and other ASD-relevant behavioral phenotypes (82–85). In addition, defects in GABA receptors, including a reduction in the number and density of GABAB and GABAA receptor subunits, have also been found in postmortem brain tissues from many patients with ASD (86). In conclusion, GABA receptor dysfunction is an important pathological mechanism in some children with ASD. Based on the facts described above, we boldly infer that a large number of patients with ASD also have dysfunction in the GABAB pathway during the fetal period. Our results indicated that the activation of the GABAB receptor by prenatal baclofen treatment exerts a beneficial effect on fetal neurodevelopment in F2 mice, which may compensate for epigenetic and VPA exposure-induced molecular perturbations. The results supported the hypothesis that therapeutic strategies designed to enhance inhibitory synaptic transmission during pregnancy and early in life may improve symptoms associated with the autism diagnosis in some children with ASD.

While the experimental results are encouraging, the current study has limitations. The complex genetic and environmental conditions of real children with ASD are impossible to replicate in animals due to the homogeneity between experimental animals and exposure to the same experimental environment. Meanwhile, the determination of whether the drug baclofen will exert adverse effects on the fetus when administered during pregnancy is difficult, although no obvious abnormalities were observed in this study. However, the results show the possibility of early intervention with GABAB receptor agonists for the treatment of children with ASD. These results are undoubtedly encouraging. In addition, our results also support the hypothesis that GABABR is a promising drug target for the treatment of neuropsychiatric disorders and developmental disorders (78, 87), which may be an important direction for the development of new drugs for ASD treatment.

## Data Availability Statement

The raw data supporting the conclusions of this article will be made available by the authors, without undue reservation.

## Ethics Statement

The animal study was reviewed and approved by the Institutional Animal Care and Use Committee of Ningxia Medical University (IACUC Animal Use Certificate No 2019-152).

## Author Contributions

FW and TS are accountable for all aspects of the work in ensuring that questions related to the accuracy or integrity of any part of the work are appropriately investigated and resolved. SJ, MH, and LX made major contributions to the conception, design and the acquisition, analysis, or interpretation of data for the work. A part of material preparation, data collection were performed by YS, JD, WL, BG, LW, YW, and CG. The first draft of the manuscript was written by SJ and all authors commented on previous versions of the manuscript. All authors contributed to the study conception and design. All authors contributed to the article and approved the submitted version.

## Funding

This study was supported by the National Natural Science Foundation of China (NSFC) (No. 82060261), the Key Research Project of Ningxia (No. 2018YBZD04917) and the Ningxia Hui Autonomous Region 13th Five-Year Plan Major Science and Technology Projects (Ningxia Brain Project) (No. 2016BZ07).

## Conflict of Interest

The authors declare that the research was conducted in the absence of any commercial or financial relationships that could be construed as a potential conflict of interest.

## Publisher's Note

All claims expressed in this article are solely those of the authors and do not necessarily represent those of their affiliated organizations, or those of the publisher, the editors and the reviewers. Any product that may be evaluated in this article, or claim that may be made by its manufacturer, is not guaranteed or endorsed by the publisher.

## Supplementary Material

Supplementary Figure 1 Flowchart for eliminating the “litter effect”.

Supplementary Figure 2 Flowchart for behavioral test.

Supplementary Video 1 Social interaction test(scene 2, F2-Con).

Supplementary Video 2 Novel object recognition task(scene 2, F2-Con).

Supplementary Video 3 Novel object recognition task(scene 2, F2-Int).

Supplementary Video 4 Open-field task (F2-Int).
