# Supplementary material for: Prenatal GABAB Receptor Agonist Administration Corrects the Inheritance of Autism-Like Core Behaviors in Offspring of Mice Prenatally Exposed to Valproic Acid
Source: Front Psychiatry. 2022 Apr 15;13:835993. doi: 10.3389/fpsyt.2022.835993 (PMC9051083; doi:10.3389/fpsyt.2022.835993)

Supplementary Figure 1: Flowchart for eliminating the "litter effect"

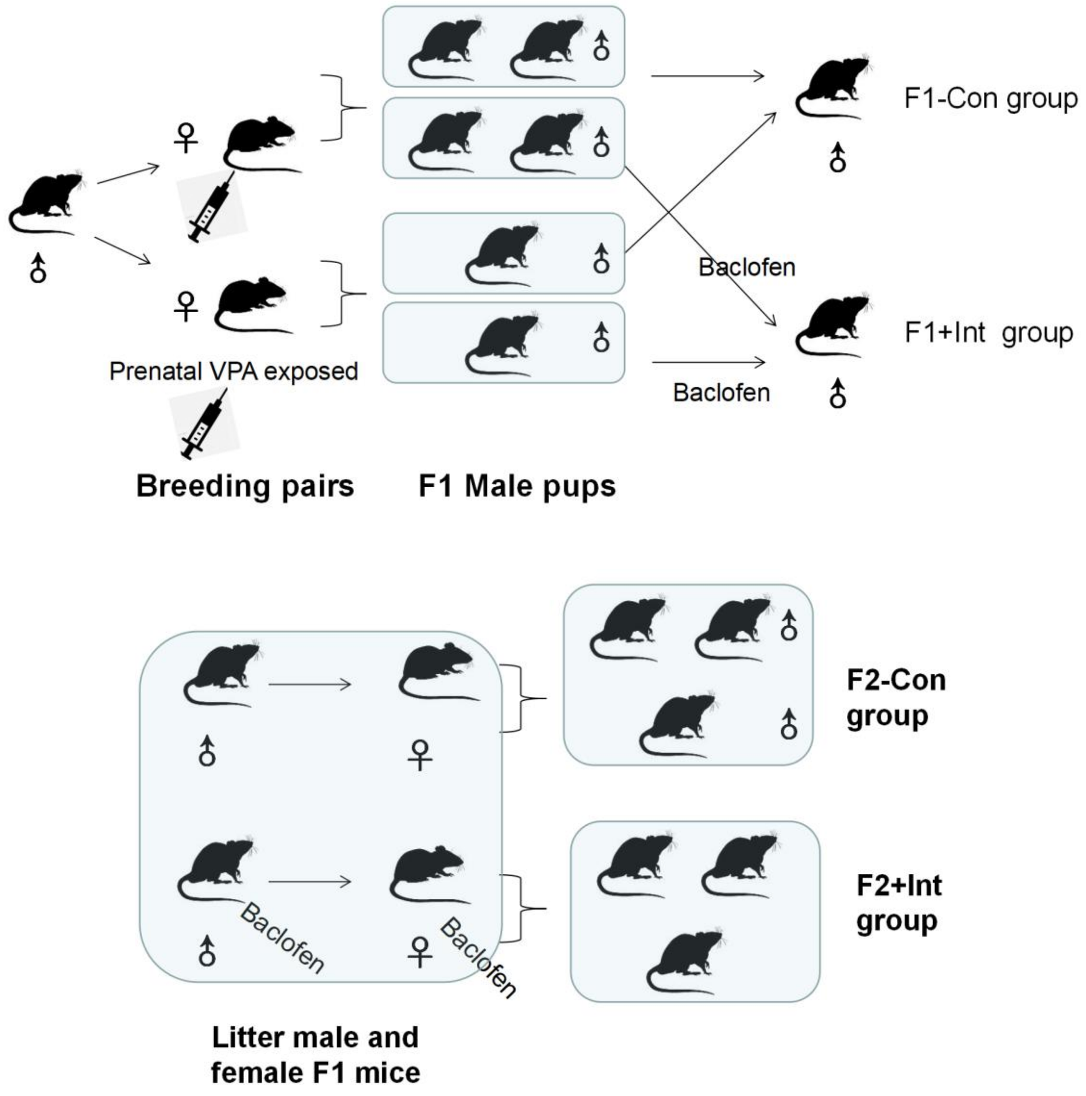

Supplementary Figure 2: Flowchart for behavioral test

①Flowchart of social interaction test

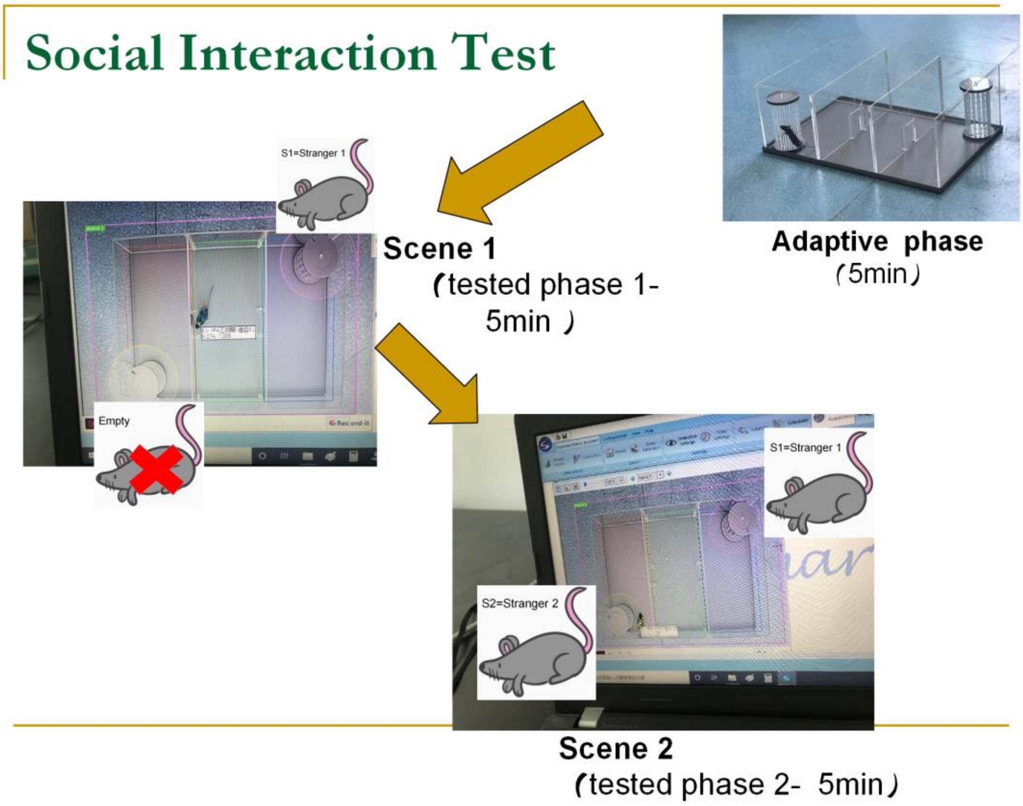

② Flowchart of novel object recognition task

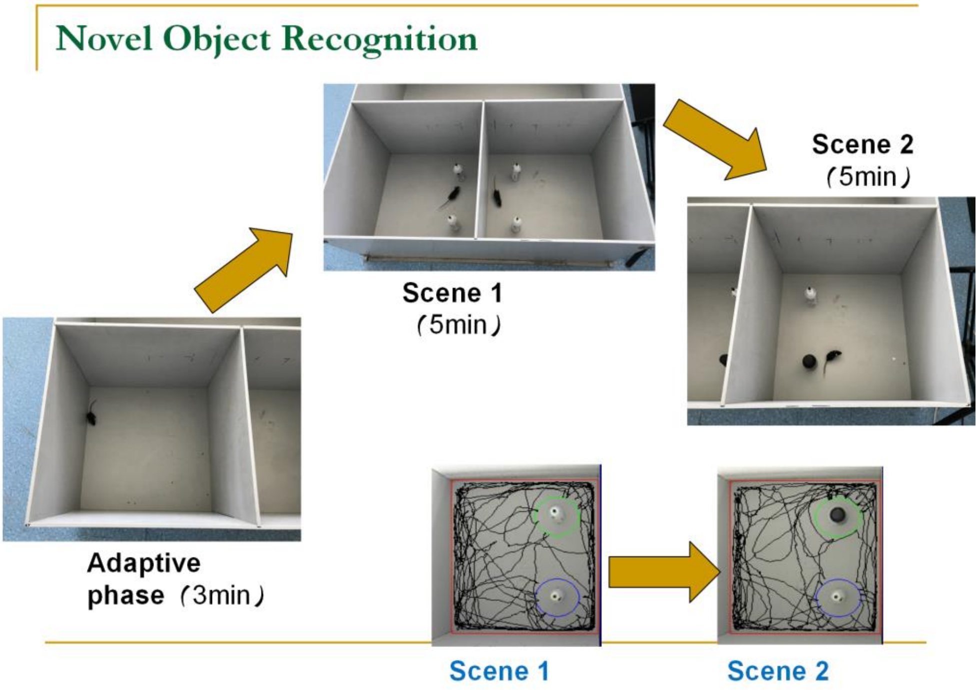

③ Flowchart of open-field task and open-field habituation task

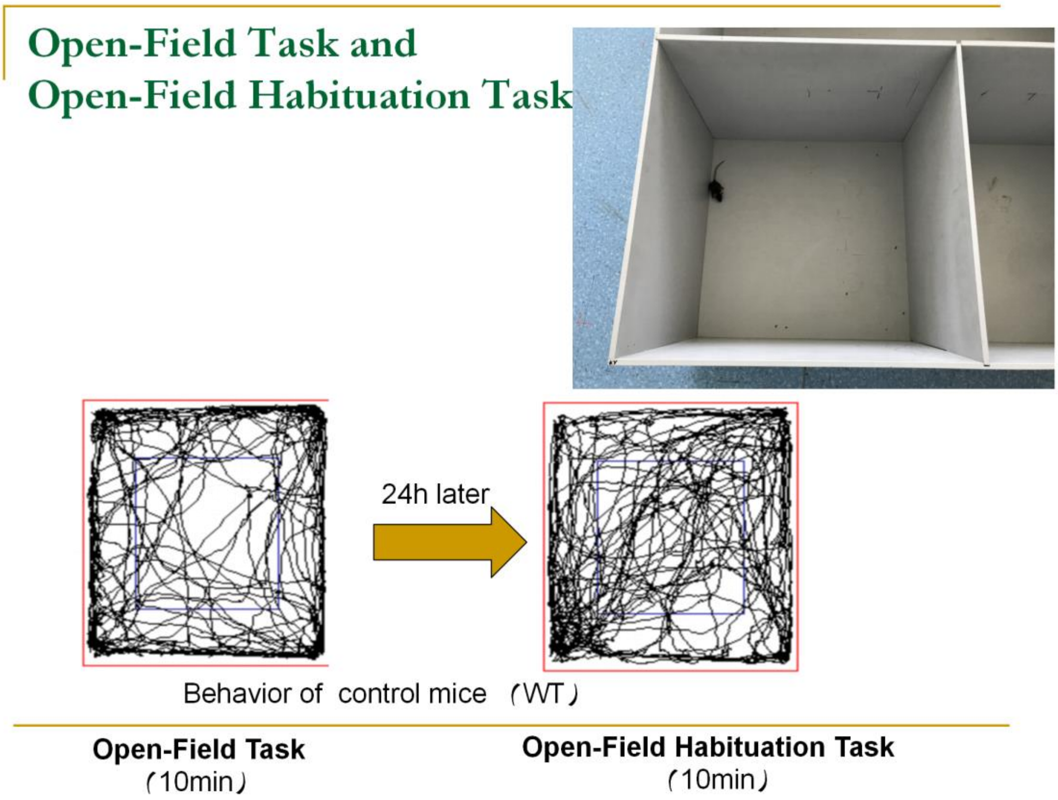

④Flowchart of marble burying test

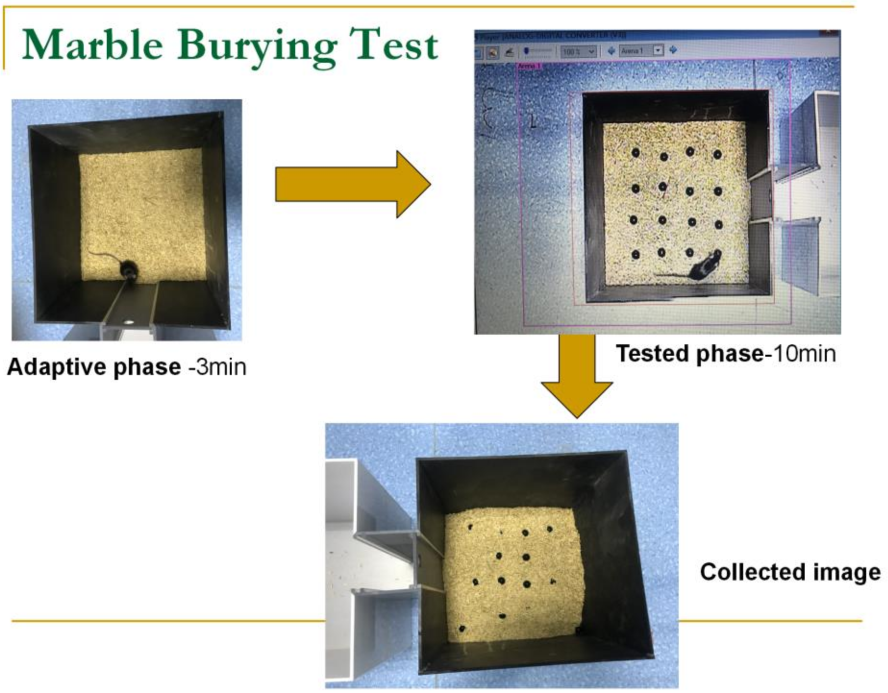

Supplement: Supplementary Figure 1 — Flowchart for eliminating the “litter effect”. [file Data_Sheet_1.pdf]
